# Who Is at Risk for Diagnostic Discrepancies? Comparison of Pre- and Postmortal Diagnoses in 1800 Patients of 3 Medical Decades in East and West Berlin

**DOI:** 10.1371/journal.pone.0037460

**Published:** 2012-05-22

**Authors:** Daniel Wittschieber, Frederick Klauschen, Anna-Christin Kimmritz, Moritz von Winterfeld, Carsten Kamphues, Hans-Joachim Scholman, Andreas Erbersdobler, Heidi Pfeiffer, Carsten Denkert, Manfred Dietel, Wilko Weichert, Jan Budczies, Albrecht Stenzinger

**Affiliations:** 1 Institute of Pathology, Charité University Hospital, Berlin, Germany; 2 Institute of Legal Medicine, University Hospital Münster, Münster, Germany; 3 Institute of Pathology, University Hospital Heidelberg, Heidelberg, Germany; 4 Institute of Pathology, University Hospital Rostock, Rostock, Germany; 5 Department of General, Visceral and Transplantation Surgery, Charité University Hospital, Berlin, Germany; University of Barcelona, Spain

## Abstract

**Background:**

Autopsy rates in Western countries consistently decline to an average of <5%, although clinical autopsies represent a reasonable tool for quality control in hospitals, medically and economically. Comparing pre- and postmortal diagnoses, diagnostic discrepancies as uncovered by clinical autopsies supply crucial information on how to improve clinical treatment. The study aimed at analyzing current diagnostic discrepancy rates, investigating their influencing factors and identifying risk profiles of patients that could be affected by a diagnostic discrepancy.

**Methods and Findings:**

Of all adult autopsy cases of the Charité Institute of Pathology from the years 1988, 1993, 1998, 2003 and 2008, the pre- and postmortal diagnoses and all demographic data were analyzed retrospectively. Based on power analysis, 1,800 cases were randomly selected to perform discrepancy classification (class I-VI) according to modified Goldman criteria. The rate of discrepancies in major diagnoses (class I) was 10.7% (95% CI: 7.7%–14.7%) in 2008 representing a reduction by 15.1%. Subgroup analysis revealed several influencing factors to significantly correlate with the discrepancy rate. Cardiovascular diseases had the highest frequency among class-I-discrepancies. Comparing the 1988-data of East- and West-Berlin, no significant differences were found in diagnostic discrepancies despite an autopsy rate differing by nearly 50%. A risk profile analysis visualized by intuitive heatmaps revealed a significantly high discrepancy rate in patients treated in low or intermediate care units at community hospitals. In this collective, patients with genitourinary/renal or infectious diseases were at particularly high risk.

**Conclusions:**

This is the current largest and most comprehensive study on diagnostic discrepancies worldwide. Our well-powered analysis revealed a significant rate of class-I-discrepancies indicating that autopsies are still of value. The identified risk profiles may aid both pathologists and clinicians to identify patients at increased risk for a discrepant diagnosis and possibly suboptimal treatment intra vitam.

## Introduction

Although clinical non-forensic autopsies are periodically considered by both clinicians and pathologists to be of high value [1]–[3] and are even supported by the vast majority of the population [4], [5], autopsy rates in the USA and in Europe have consistently declined during the last decades: from about 60% in the USA in the 1950’s to <5% in the first years of the 21^st^ century [6], [7]. Consistently, a representative poll of the German National Professional Organisation of Pathologists (Berufsverband Deutscher Pathologen) estimated an autopsy rate of approximately 3.5% in Germany for 2004 [8]. The reasons are manifold and comprise, amongst others, financial interests/reimbursement policy, political disinterests, concerns over litigation and deficient communication between clinicians, relatives and pathologists, respectively as well as the often privately quoted notion that there is no demand for clinical autopsies due to improved diagnostic techniques rendering autopsies redundant [7], [9]–[11]. However, it is well known that causes of death ascertained by the attending clinicians disagree in a considerable rate with the pathological findings in clinical autopsies [2], [12]–[15]. Two studies comparing clinical diagnoses with autopsy findings revealed even no improvement in diagnostic concordance over time [16], [17].

A study initiated by the U.S. Department of Health and Human Services in 2002 evaluated more than 30,000 entries from Medline and Cochrane literature searches and concluded that only 25% of the causes of death statements in death certificates are correct and clinicians are not able to predict which autopsies will be of high diagnostic yield [18]. Moreover, the major discrepancy rate between clinical and pathological diagnoses was 35.4%. The German Medical Association (Bundesärztekammer) published a similar paper in 2005 drawing comparable conclusions [19].

In this context, it is suggested that clinical autopsies are a reliable and economically reasonable quality control measure in hospitals [20]–[23].

However, recent comprehensive data from a large European patient cohort encompassing both university and community hospitals are lacking. Hence, we set out to investigate the rate and significance of major and minor diagnostic discrepancies between clinical and autopsy diagnoses in 1,800 randomly selected adult patients treated at the Charité University Hospital Berlin and at community hospitals in Berlin and Brandenburg over three decades from 1988 to 2008. These data were correlated with various parameters including patient demographics, disease groups, hospital type, clinical subspecialty and type of ward.

In addition to evaluating the accuracy of clinical diagnoses with respect to autopsy results, our analysis also allowed us to deduce diagnosis-specific discrepancy statistics. From these results risk profiles can be derived that may help to identify patients with a higher probability to receive a discrepant major diagnosis intra vitam. These data may also help to define a subgroup of patients, for which the autopsy will particularly contribute to an improved understanding of the clinical course of the patient including the circumstances of death.

Moreover, our study also aims to shed light on the influence of two different health care systems at the very end of the cold war by comparing the discrepancy rate in university and community hospitals of and around East and West Berlin in 1988 – one year prior to the fall of the Berlin wall.

## Methods

### Ethics Statement

This register-based research study of pre-existing personal data has been approved by the Ethical Committee of the Charité University Hospital Berlin, Germany and meets the German legal requirements concerning human subjects (Application No.:E A4/1071/11).

### Data Acquisition and Analysis of Autopsy Reports

Excluding neonates, children and adolescents (until the age of 18), each autopsy case of the Institute of Pathology of the Charité University Medicine (Campus Charité Mitte (CCM) and Campus Virchow Hospital (CVK)), Berlin, Germany, from 1988, 1993, 1998, 2003 and 2008 was analyzed retrospectively with permission of the local ethics committee (Application No.:E A4/1071/11). From these 3,299 cases, all clinical and pathologic-anatomical diagnoses were recorded as well as the cause of death, age, sex, and the location of the last treatment (table 1) including the type of hospital (university or community hospitals), clinical subspeciality and type of ward (intensive care unit (ICU) and low and intermediate care unit (LAICU)).

**Table 1:** Clinical information and meta data about autopsy patients from 1988 to 2008.

Characteristics	1988 (GDR)		1988 (FRG)		1993		1998		2003		2008		Total	
*General Data*	all	study	all	study	all	study	all	study	all	study	all	study	all	study
n (pat.)	1198	300	305	300	387	300	537	300	482	300	390	300	3299	1800
male [%]	55.0	58.0	40.7	40.7	55.8	56.7	57.2	57.3	62.4	61.3	59.0	60.3	55.7	55.7
female [%]	45.0	42.0	59.3	59.3	44.2	43.3	42.8	42.7	37.6	38.7	41.0	39.7	44.3	44.3
age (mean/median)	61.662.0	62.262.5	72.876.0	72.876.0	61.162.0	61.562.0	63.665.0	63.264.0	62.364.0	62.864.5	63.766.0	63.865.0	63.364.0	64.466.0
***Autopsy rates***														
university hospitals [%]	96.6		44.5		22.0		22.2		26.2		20.1			
community hospitals [%]	77.0		19.6		11.7		6.3		6.2		5.3			
***Location of last treatment (gross)***														
university hospitals [%]	45.9	43.6	58.0	58.7	62.3	62.0	56.7	56.5	68.7	67.0	61.5	62.0	55.9	58.3
community hospitals [%]	54.1	56.4	41.6	41.3	37.7	38.0	43.3	43.5	31.3	33.0	38.4	38.0	44.1	41.7
intensive care unit [%]	24.2	25.0	3.6	3.7	32.0	33.3	35.8	33.3	39.8	40.0	65.9	66.7	36.2	33.7
low and intermediate care unit [%]	72.9	70.7	0.3	0.3	66.9	65.7	60.5	62.3	60.0	59.7	34.1	33.3	63.8	48.7
***Location of last treatment (detailed)***														
(internal) medicine [%]	47.3	41.7	58.7	58.7	55.8	53.7	43.0	43.3	50.4	50.3	44.9	45.3	48.8	48.8
surgery (incl. cardiac, neurol., bone) [%]	18.3	21.3	19.3	19.3	11.4	11.3	27.0	26.7	28.4	28.7	35.4	35.7	22.5	23.8
anaesthesiology [%]	13.4	12.3	1.0	1.0	19.6	21.3	16.0	14.0	8.9	10.3	14.4	13.7	12.9	12.1
neurology [%]	3.3	2.7	0.7	0.7	4.4	4.3	3.5	4.7	2.3	1.7	3.8	3.7	3.1	2.9
other [%]	16.7	20.3	18.7	18.7	8.0	8.3	6.3	6.7	10.0	9.0	1.3	1.3	11.4	10.7
unknown [%]	1.1	1.7	1.6	1.7	0.8	1.0	4.1	4.7	0.0	0.0	0.3	0.3	1.3	1.6

The data presented here do not reveal significant differences between “all” and “study” group concerning the distribution of the characteristics considered, therefore representativeness can be assumed.

The Charité University Medicine Berlin is a joint institution of the Humboldt University Berlin and Free University Berlin and comprises tertiary care hospitals at several locations (campi) within the city. Due to the diagnostic responsibilities of the Institute of Pathology that covered the campi CCM and CVK at the time point when the study was launched, cases from these two campi were included.

Community hospitals are defined as primary and secondary care facilities. They are located in the area of Berlin/Brandenburg and do not directly belong to the Charité University Hospital. For the period investigated, 28 different community hospitals in the Berlin districts Charlottenburg-Wilmersdorf, Mitte, Pankow, Reinickendorf, Spandau, Steglitz-Zehlendorf, Treptow-Köpenick as well as 8 cities of the adjacent Federal State of Brandenburg were included.

Clinical diagnoses were those listed by the clinician on the autopsy request form. The latter requires the clinician to give both, a brief present and past medical history and a clinically estimated causal sequence of death (e.g. deep vein thrombosis ➔ pulmonary thrombembolism ➔ acute right heart failure). Pathological diagnoses were those listed on the final autopsy report. All cases investigated comprise patients who underwent a complete pathological autopsy, including histological assessment of all major internal organs. Autopsies were performed by at least one experienced in-house pathologists from the Campus Charité Mitte (CCM; in 1993, 1998, 2003 and 2008 also supplying the CVK) or the Campus Virchow Hospital (CVK; in 1988 still part of the Free University, Berlin-West). Each autopsy case was both macroscopically and microscopically reviewed by a consultant ( = senior pathologist) of the same institution. Pathological diagnoses were grouped into seven disease groups according to the International Classification of Diseases, 10th edition (ICD-10): Certain Infectious and parasitic diseases (A00–B99, INF), neoplasms (C00–D48, NP), diseases of the circulatory system (I00–I99, CV), diseases of the respiratory system (J00–J99, PU), diseases of the digestive system (K00–K93, GI), diseases of the genitourinary/renal system (N00–N99, RE) and miscellaneous (remaining diagnoses, MISC).

### Sample Size Calculation

A sample size calculation was carried out in order to estimate the number of patients that are needed to detect significant changes in diagnostic discrepancies. Two different kinds of objectives were taken into account: First, detection of linear changes in a time series (1988 to 2008) and, second, detection of changes between two groups of patients. Monte-Carlo simulations were executed in order to estimate the power of the test for an increasing sample size. In short, for a fixed number of samples, 10,000 distributions of proportions were drawn from the binomial distribution. The test was executed for each of the distributions and the power was estimated by counting the number of significant tests. This procedure was repeated for increasing numbers of samples until the power we aimed at was reached.

It is expected that the diagnostic accuracy improved between 1988 and 2008 and led to a substantial decrease of discrepancy rates during these years. Hence, the study was powered high enough to detect a 2.5% decrease per year resulting in the following sample size estimation: The number of patients that is needed to detect a linear tend of 40%, 37.5%, 35%, 32.5%, 30% in a time series 1988, 1993, 1998, 2003, 2008 with significance equals to 0.05 and power equals to 0.8 is n = 287 for each year. This number of samples will yield even higher power for time series with decrement 2.5% that start at a lower percentage, e.g. for the series 25%, 22.5%, 20%, 17.5%, 15% (in this case the number of samples needed is n = 199 for each year).

Additionally, we aimed at comparing the discrepancy rates between subgroups of patients, for instance, patients with diseases of group A (e.g. cardiovascular diseases) and patients with diseases of group B (e.g. neoplastic diseases) or patients treated in university hospitals and patients treated in community hospitals. For this purpose, we demanded the study to be powered highly enough to detect a 10% change in discrepancy rates between two subgroups of patients and obtained the following sample size estimation: The number of patients needed to distinguish between 55% and 45% in discrepancy in two patient groups with significance 0.05 and power 0.8 is n = 407 for each of the groups. This number of samples will yield even higher power to distinguish between other differences like 45% and 35%, 35% and 25%, 25% and 15%, 10% and 5% and so on. To attain the necessary number of 407 patients for subgroup comparison, patients were pooled, e.g. over the years 1993–2008 after the German reunification.

### Randomization and Classification of Discrepancy Classes

Based on the sample size calculations, from a total of 3,299 reviewed autopsied patients, 300 cases were randomly selected for each of the years 1993, 1998, 2003 and 2008. For 1988, 600 autopsy cases were randomized comprising East Berlin (n = 300; Institute of Pathology, CCM) and West Berlin (n = 300; Institute of Pathology, CVK; at that time part of the Free University, West Berlin).

Subsequently, all randomized cases (n = 1,800) were assessed with regard to discrepancies between clinical and pathological diagnoses. Discrepancies were graduated into six different classes according to the modified Goldman criteria [2], [13], [16] (table 2). In brief, a major discrepancy was assigned when the discrepancy in a diagnosis was related to the cause of death (class I and II). Minor discrepancies comprise discrepancies in diagnoses with no direct relation to cause of death (class III and IV). Cases were assigned to the class with the most severe discrepancy. Cases without discrepant diagnoses were grouped into class V and non-classifiable cases (due to i.e. missing data or medicolegal background) were designated as class VI. 

**Table 2:** Criteria for evaluating discrepancies modified after Goldman et al. (1983) and Battle et al. (1987).

major discrepancies	class I	discrepancies in major diagnoses with relation to cause of death
		detection would have led to changes in management and therapy
		detection and adjusted therapy **could have prolonged** survival or cured the patient
	class II	discrepancies in major diagnoses with relation to cause of death
		detection would have led to changes in management and therapy
		detection and adjusted therapy **would not have prolonged** survival or cured the patient
minor discrepancies	class III	discrepancies in minor diagnoses with no direct relation to cause of death
		Symptoms should have been treated or would have eventually affected prognosis
	class IV	discrepancies in minor diagnoses with no direct relation to cause of death
		Non-diagnosable (occult) diseases with possible genetic or epidemiological importance
other	class V	no discrepancies
	class VI	non-classifiable cases

Discrepancies were classified by two pathologists (DW and AS) with ample autopsy experience. In cases of no agreement, a senior pathologist (HJS, AE, CD or WW) was consulted. Additionally, clinically unclear and difficult cases were analyzed by a board-certified physician from the Department of General, Visceral and Transplantation Surgery, Berlin, Germany (CK).

As result, each case was assigned unambiguously to one of the classes I–VI.

### Statistical Analysis

Data analysis was performed using the statistical language R. Statistical significance of 2×2 tables was assessed by Fisher’s exact test. Significance of a trend in time series was assessed by the Cochran-Armitage test as it is implemented by function prop.trend.test() in the R package stats.

In order to identify factors for a high risk of discrepancy, we have combined an extensive subgroup analysis with heatmap visualization. To exclude statistical bias due to different health care systems, we considered the data of the 1,200 cases that were autopsied after the German reunification (1993–2008). In detail, patients were grouped according to demographics or the ward where they were treated. This grouping was intersected with a second grouping according to disease groups. For each of the subgroups, the percentage of discrepancies was calculated and compared to the percentage of discrepancies for patients not in the subgroup. Significance of a decreased or increased discrepancy risk was assessed by Fisher’s exact test.

In general, significance assessments were based on two-sided p-values. P-values <0.05 were considered as significant, p-values <0.1 as borderline significant.

## Results

### Analysis of the Autopsy Patient Meta Data

A total of 3,299 autopsy cases of the Charité Institute of Pathology between 1988 and 2008 were recorded (table 1, referred to as “all” groups). With the exception of the year 1988 (West Berlin), between 1988 and 2008 more autopsies were performed on men than on women (1.3 :1). In university hospitals the autopsy rate remained nearly constant at a mean value of about 23% between 1993 and 2008. Before reunification in 1988, the autopsy rate was considerably higher in East (96.6%) versus West Berlin (44.5%). A similar trend was seen for the autopsy rates of community hospitals where the autopsy rate decreased from 77.0% (GDR [German Democratic Republic], 1988) and 19.6% (FRG [Federal Republic of Germany], 1988) to a constantly low rate of about 6% (1998 to 2008). The proportion of autopsied patients from university hospitals continuously increased from 45.7% (1988) to 87.2% (2008) in the course of time, whereas the correspondent proportion of autopsies requested by community hospitals declined from 54.0% (1988) to 12.8% (2008). Noteworthy, the proportion of autopsied patients under intensive care before death strongly increased over time from 24.2% (1988) to 65.9% (2008). Similar results were found for the study population, which showed an increase of ICU cases from 25.0% (1988) to 66.7% in 2008. Autopsies of patients from low and intermediate care units (LAICUs), however, declined from 72.9% (1988) to 34.1% (2008). During the time period investigated, patients were most commonly treated on a ward of (internal) medicine, followed by surgical wards. Cardiovascular diseases were the most frequent diagnoses according to autopsy, followed by neoplastic and pulmonary diseases (table 3, “all” groups). With respect to the cause of death, cardiovascular diseases ranked first, followed by neoplastic diseases (table 3, “c.o.d.”).

**Table 3:** Distribution of pathological diagnoses at autopsy.

	1988 (GDR)			1988 (FRG)			1993			1998			2003			2008			Total		
	all	study	c.o.d.	all	study	c.o.d.	all	study	c.o.d.	all	study	c.o.d.	all	study	c.o.d.	all	study	c.o.d.	all	study	c.o.d.
n (pat.) = 100%	1198	300	300	305	300	300	387	300	300	537	300	300	482	300	300	390	300	300	3299	1800	1800
CV [%]	61.0	61.3	39.7	93.1	93.0	42.3	60.5	60.0	40.7	83.8	82.3	42.3	90.5	92.7	43.3	85.6	85.7	46.3	74.8	79.2	42.4
NP [%]	45.3	45.3	31.3	31.5	31.7	20.3	35.9	34.7	24.3	41.7	42.7	28.0	36.5	34.3	23.0	33.6	32.0	15.0	39.7	36.8	23.7
PU [%]	28.4	29.3	0.3	58.0	58.0	0.0	22.2	23.0	0.0	36.5	36.7	0.0	38.8	39.7	0.0	40.0	40.3	0.0	34.6	37.8	0.1
RE [%]	6.4	7.3	1.0	18.4	18.7	4.0	4.7	5.3	1.3	3.9	4.3	0.3	6.6	7.0	0.0	7.4	7.7	0.0	7.1	8.4	1.1
INF [%]	2.8	3.7	2.3	5.6	5.7	3.0	5.2	6.0	3.0	9.5	12.0	8.0	12.3	13.6	10.3	7.9	9.7	8.0	6.4	8.5	5.8
GI [%]	22.0	21.7	9.7	35.4	35.0	6.0	28.7	29.0	15.7	20.3	18.7	5.0	24.7	25.7	6.0	30.5	33.0	8.7	25.2	27.2	8.5
MISC [%]	25.0	21.7	0.7	51.1	51.7	1.3	27.7	27.3	1.7	30.5	30.3	2.0	27.2	27.0	3.0	29.2	30.0	8.0	29.4	31.3	2.8

“All” comprises the total number of cases investigated from the respective year. The “study” group of a year relates to the cohort randomly selected from the “all” group in order to determine the discrepancy rate. The data presented here do not reveal significant differences between “all” and “study” group concerning the distribution of post-mortem diagnoses, therefore representativeness can be assumed. “c.o.d.” shows the distribution of causes of death of the “study” group with regard to the disease groups considered.

Disease groups according to ICD-10: CV – cardiovascular, NP – neoplastic, PU – pulmonary, RE – renal/genitourinary, INF – infectious, GI – gastrointestinal, MISC – miscellaneous.

### Assessment of Discrepancy Rates by Means of Modified Goldman Criteria

Comparison of the study population with the total cohort (referred to as “study” and “all”, respectively, see table 1 and 3), reveals that the 1,800 randomly selected cases were representative for the total cohort of 3,299 autopsy cases.

**Figure 1:**
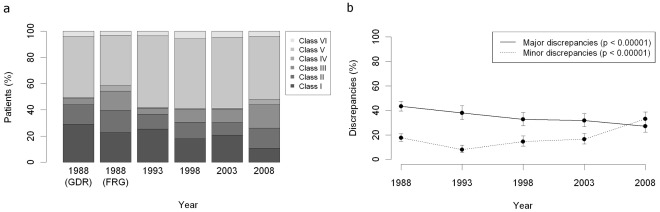
Development of the discrepancies between clinical and pathological diagnoses at the Charité hospital during the last 20 years. 300 cases were included into the analysis for each of the years 1988 (GDR), 1988 (FRG), 1993, 1998, 2003 and 2008. Cases were assigned to discrepancy classes I to VI according to the modified Goldman criteria. a) Country and time dependent distribution of the discrepancy classes I–VI. b) Significant decrease of major discrepancies and significant increase of minor discrepancies between 1988 and 2008. 1988 =  pooled GDR and FRG cases.

The randomized cases were assigned to classes I to VI according to the modified Goldman criteria (table 2). Figure 1a shows the country and time dependent distribution of the discrepancy classes. Herein, the proportion of class I discrepancies significantly declined by 15.1% from 25.8% (1988) to 10.7% (2008) (p<0.00001, 95% CI: 7.7%–14.7%). The proportion of class III discrepancies increased from 13.7% (1988) to 27.0% (2008) (p<0.00001). There was no significant change in the proportions of class II and class IV discrepancies. The largest group comprised the cases without discrepancies (49.1%, class V), whereas only a small number of cases (4.1%, class VI) were non-classifiable.

The temporal development of the total major discrepancy rate (class I and II) reveals a significant decline by 16.3% from 43.4% (1988) to 27.1% (2008) (figure 1b, p<0.00001). Minor discrepancies (class III and IV) increased from 16.4% (1988) to 33.0% (2008) (p<0.00001).

### Investigation of Factors Possibly Influencing the Class I Discrepancy Rate

#### 1.) Sex and Age

**Figure 2:**
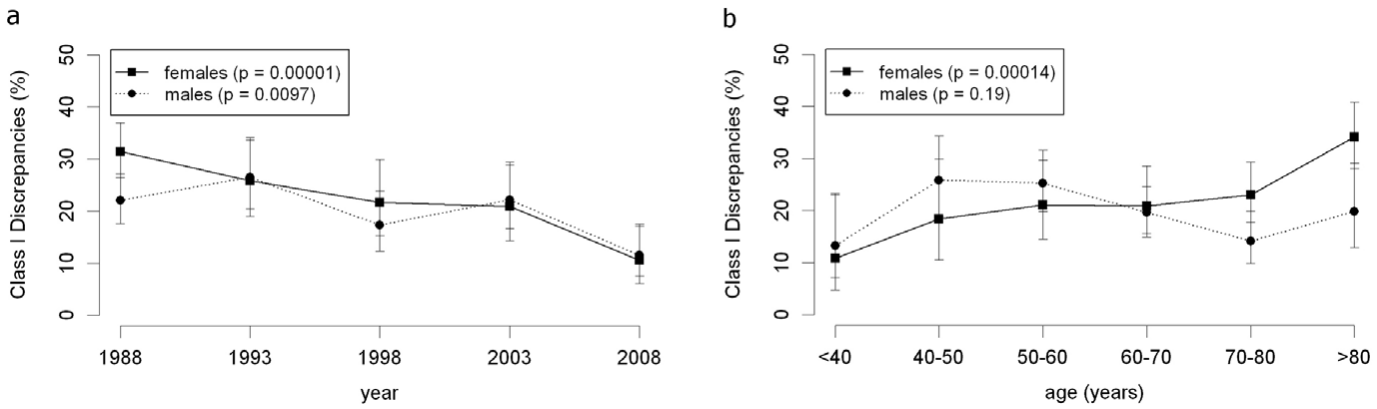
The influence of age and sex. a) Class I discrepancy rates for male and female patients. b) Dependence of the class I discrepancy rates on age and sex of the patients. All 1800 “study” cases (1988–2008) were included into the analysis.

Class I discrepancies of female patients significantly decreased from 31.4% (1988) to 10.5% (2008) (figure 2a, p<0.00001). The decrease of class I discrepancies of male patients from 22.0% (1988) to 11.5% (2008) was also significant (p = 0.0097).

In 1988, the class I discrepancy rate of female patients was 31.4% compared to 22.0% for male patients (borderline significant, p = 0.057). From 1993 to 2008 the class I discrepancy rate did not significantly differ between male and female patients.

Class I discrepancies of female patients significantly increased with age (p = 0.00014), whereas no significant change was observed for male patients (p = 0.19; figure 2b).

#### 2.) Type of hospital

**Figure 3:**
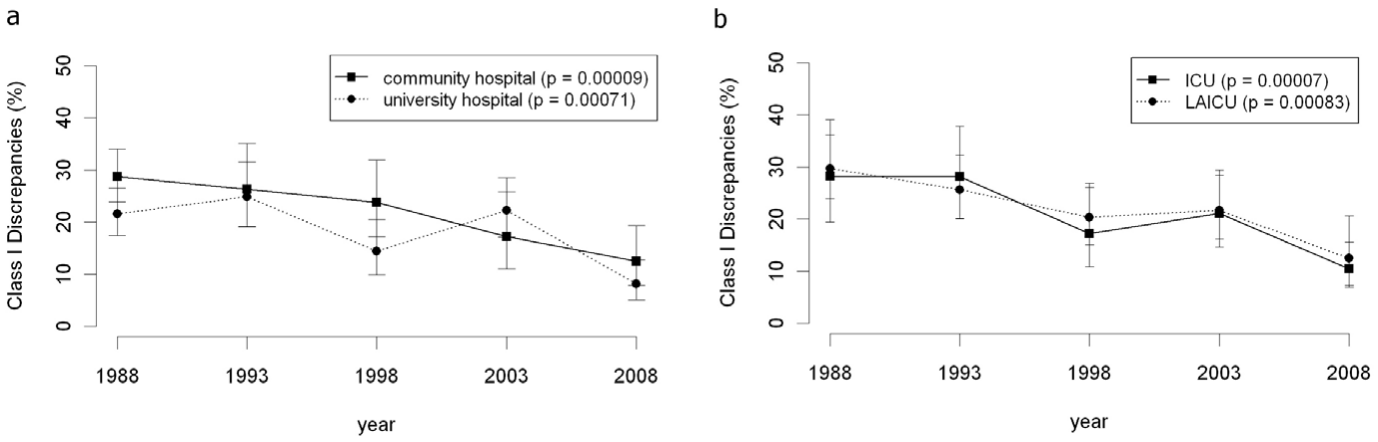
Influence of type of hospital and type of ward. a) Class I discrepancy rates for university and community hospitals. b) Class I discrepancy rates rates for ICU’s and LAICU’s.

From 1988 to 2008 the class I discrepancy rate was significantly higher in community hospitals than in university hospitals (p = 0.045) (figure 3a). In university hospitals the class I discrepancy rate continuously declined (p = 0.00071). In particular, in 2008 university hospitals (5.4% class I discrepancies) significantly outperformed the community hospitals (14.7% class I discrepancies, p = 0.0082).

#### 3.) Type of ward

The class I discrepancy rate of both patients from ICUs and from LAICUs significantly declined from 1988 to 2008 (p = 0.00007, p = 0.00083; figure 3b). No significant difference was found in comparing the rates against each other.

#### 4.) Clinical subspecialty

Internal, surgical, anaesthesiological and neurological wards initiated the vast majority of autopsies in Berlin between 1988 and 2008 (table 1).

Of these (figure S1), internal (p = 0.001), surgical (p = 0.00042), and anaesthesiological wards (p = 0.015) showed a significant improvement of the class I discrepancy rate over the years investigated. Neurological wards, however, kept their class I discrepancy rate nearly constant (p = 0.41).

#### 5.) Disease group (according to ICD-10)

For each of the disease groups we calculated the proportion of class I discrepancies in relation to the total number of patients within a disease subgroup. We observed that cardiovascular, neoplastic, pulmonary and gastrointestinal diseased patients showed a significant decline in class I discrepancies of their respective disease group over time (figure S2 a,b,c,d,), e.g. among cardiovascular diseased patients a decrease of class I discrepancies from 26.9% in 1988 to 11.8% in 2008 (p = 0.00032). Regarding the other disease groups, no significant trend was found in changes of the class I discrepancy rates over time (data not shown).

Next, we calculated the proportion of the patients with a class I discrepancy in relation to the total number of patients dying from a disease group. Only cardiovascular causes of death showed a significant decline in class I discrepancies of their respective disease group over time (figure S3 a), whereas in the other disease groups the causes of death revealed no significant trend over time (figure S3 b,c,d and data not shown).

### Outline of Patients’ Risk Profiles for Receiving a Discrepant Major Diagnosis (Class I)

**Figure 4:**
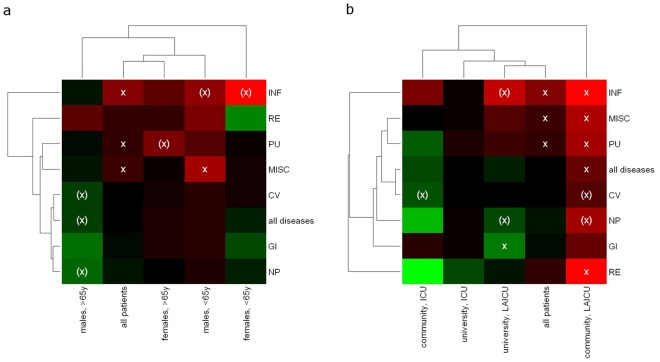
Identification of low and high risks case characteristics for class I discrepancies based on a two-dimensional subgroup analysis. Patients were grouped according to demographics or the ward where they were treated. This grouping was intersected with a second grouping according to disease groups. For each of the subgroups, the percentage of discrepancies was calculated and compared to the percentage of discrepancies for patients not in the subgroup. Heatmaps of discrepancy rates were generated using hierarchical clustering based on the euclidean distance of rate profiles. The overall class I discrepancy rate of 19.5% [17.4%–21.9%] for 1200 cases (black) between 1993 and 2008 served as reference for decreased (green) or increased (red) class I discrepancy rates. a) Dependence of class I discrepancy rates on ICD-10 disease groups, age and sex of the patients. Sample sizes were comparable for patients being under 65 years (n = 615) and patients older than 65 (n = 585). b) Dependence of class I discrepancy rates on ICD-10 disease groups, type of hospital and type of ward. x  =  p<0.05 (x)  =  p<0.1.

In order to identify cases with increased risk for class I discrepancies, heatmaps of discrepancy rates were generated (figure 4a,b). The overall class I discrepancy rate of 19.5% [17.4%–21.9%] for 1,200 cases (black) served as reference.

As shown in the last column of figure 4b, we generally detected significantly increased class I discrepancy rates in patients treated at community hospitals on a low and intermediate care unit (LAICU). For this particular patient collective, most class I discrepancies were found in cases with genitourinary/renal (53.8%, p = 0.033) or infectious (47.4%, p = 0.033) diseases. Furthermore, female patients under the age of 65 with an infectious disease (47.1%, p = 0.05) showed a borderline-significant increase in the class I discrepancy rate.

The significantly lowest class I discrepancy rate was found in cases from university hospitals, treated at a LAICU and diagnosed with a gastrointestinal disease (10.0%, p = 0.031).

### Comparison of Discrepancies in East and West Berlin in 1988

No statistical significant differences were found for the class I discrepancy rate comparing the total study groups of East (n = 300) and West Berlin (n = 300) data in 1988 (figure 1a, 30.2% versus 23.4%, p>0.1).

Subgroup analyses regarding sex, age, type of hospital, clinical subspecialty and disease groups were performed in order to compare the class I discrepancy rates in East and West Berlin in 1988. Concerning sex and age no statistically significant differences between East and West were found. Likewise, the comparison of class I discrepancy rates did not show any significant differences with respect to the type of hospital as well as to the clinical subspecialty. Furthermore, subgroup analyses of the disease groups did not reveal significantly different class I discrepancy rates between East and West Berlin.

The same results with no statistical differences regarding the total groups and the subgroup analyses were found for the major discrepancy rate (class I and II combined) (54.2% versus 59.1%, p>0.1).

## Discussion

This well-powered retrospective autopsy study comprising the largest current case series from both community and university hospitals reveals a reduction over time in the frequency of major discrepancies. This held particularly true for class I discrepancies between clinical and autopsy diagnoses. Our results based on 1,800 cases of three medical decades in East and West Berlin are consistent with the data presented by Shojania et al. (2002) [18] and a meta-analysis also conducted by Shojania et al. in 2003 [3]. Shojania et al. (2002) reported class I and class II discrepancies of 10.2% and 25.2%, respectively in a study initiated by the US Department of Health and Human Services that evaluated approximately 35,000 literature entries in Medline and Cochrane data base. Analyzing 53 autopsy studies from 1966–2002, Shojania and colleagues (2003) found a range for major discrepancies from 4.1% to 49.8% (median 23.5%) and class I discrepancies ranging from 0 to 20.7% (median 9.0%), respectively.

This is in line with our data data, which show only slightly higher discrepancies (in 2008 10.7% for class I discrepancies and 27.1% for combined major discrepancies (median values) and a comparable relative reduction by 15.1% for class I discrepancies from 1988 to 2008. The strong decline of major discrepancies over the last decades, however, is consistent with recent data and disagrees with other studies [16], [17] reporting no decrease in discrepancies over time.

The slightly higher frequency of class I and II discrepancies in our study may be explained on the one hand by the fact that clinical information was, as in many other studies [15], [16], [24]–[26], solely based on the data on the autopsy request form and not on an extensive medical chart review (as conducted by Sonderegger-Iseli et al. 2000 [2]) thereby possibly slightly overestimating the rate of major discrepancies due to a potential loss of clinical information in single cases. However, this does not imply that the clinical data actually given on the request form, which include a rather comprehensive past and present medical history for every case as well as the causal sequence of death, are of limited validity or reliability.

On the other hand it is also agreed [2], [16] that the definition of the respective discrepancy classes does not always allow for a clear-cut assignment of the cases since diseases and its influence regarding the outcome of the patient are, especially in multimorbid patients, rather continuous and interconnected than distinct. Last but not least, it has been argued that case selection leads to a certain bias since many autopsies are requested for cases with a particularly complex clinical picture often causing a diagnostic and therapeutic dilemma in which discrepancies are more likely to be observed. An intervention study could indeed show that an incremental increase in the overall autopsy rate, and thereby relatively decreasing the number of complex cases, markedly reduced the proportion of discrepant cases [24]. However, data on that issue are ambiguous. For example, Shojania and colleagues [27] argue that low autopsy rates without adjustment for the prevalence of non-autopsied deaths may even cause overestimation of the performance of ante-mortem clinical diagnoses.

We also observed a clear decrease in autopsy rates for both, university and community hospitals in the area of Berlin/Brandenburg.

Our study reveals a statistically significant increase in minor discrepancies corroborating the observations made by Sonderegger-Iseli and colleagues [2]. On the one hand, this may be due to an actual rise in minor diagnostic discrepancies. On the other hand, with the reduction of major discrepancies, minor diagnoses may be more carefully recorded by pathologists, thus leading to a certain bias.

Although we could not observe a significant correlation between sex and class I discrepancies, sex adjusted analysis, however, revealed a positive correlation of age and the frequency of class I discrepancies in female patients. These data match those described in the study of Battle et al. (1987) [13], who found a significantly higher proportion of major discrepancies in women than in men and demonstrated increasing age-dependent rates for major discrepancies.

Our study design also allowed us to determine the discrepancy rate in both community and university hospitals and revealed a significantly higher discrepancy rate for patients treated at community hospitals. Although this is in good agreement with the data from numerous other studies [12], [13], [28], [29], it has to be emphasized that in our opinion especially these autopsy cases appeared to be those with complex and multimorbid clinical pictures. Furthermore, we found the autopsy rates in community hospitals always to be lower than those in university hospitals (see table 1). Interestingly, Stevanovic and colleagues (1986) [12] described a highly significant increase of the class I discrepancy rate at Belgrade University Hospital in relation to an acute decline of the autopsy rate. Hence, as already discussed, low autopsy rates may lead to a selection bias of clinically problematic and complicated cases, which in turn increases the class I discrepancy rate of the community hospitals. Another important aspect was brought up by Battle and colleagues (1987) [13] who argued that community hospitals may often include a disproportionate number of elderly patients, which contributes to a disproportionately high class I discrepancy rate. Interestingly, we observed an increased median age of patients in community hospitals compared to university hospitals, which converge over time: 68 yrs (East Berlin 1988), vs. 55.5 yrs, 83 yrs (West Berlin 1988), vs 69 yrs, 64.5 yrs (1993) vs. 59 yrs, 66 yrs (1998) vs. 63 yrs, 63 yrs vs. 65 yrs (2003) and 67 yrs vs. 65 yrs (2008).

One may also pose the question, whether inherent infrastructural constraints, such as the limited availability of diagnostic or interventional facilities as well as a lack of certain specialities for consultative examinations, e.g. department of infectious diseases, may also account for the higher class I discrepancy rate at least in some of the community hospitals. The current economic strains in the health care system leading to limited financial and/or personnel resources may further aggravate these conditions.

Although some studies that focused on adult ICUs report a comparably higher rate of class I discrepancies [3], we did not observe a statistically significant difference regarding class I discrepancies between patients treated in the ICU and LAICU, respectively. We attribute this finding to the extensive diagnostic work-up and continuous monitoring of critically ill patients treated at ICUs, which counteracts the complex clinical picture. In fact, a large prospective study conducted by Combes et al. (2004) [30] found a class I discrepancy rate of 10.2% in 1492 patients admitted to the ICU, which matches our data on ICU cases in 2008 very well. In both studies, the autopsy rates for ICU patients in the respective years were well above 50%. As already shown by Combes and colleagues, our study mainly revealed that among the class I discrepant cases, the majority of patients were immunocompromised and frequently had associated opportunistic infections. Less frequent examples for class I discrepancies in our cohort are cases where clinical reasoning lead to the diagnosis septic shock or shock of unknown origin but autopsy revealed a high grade Non-Hodgkin lymphoma or a ruptured gastric ulcer as underlying cause of the disease. As reviewed by De Vlieger et al. [31] there is accumulating evidence matching our observations that the discrepancy rates on ICUs can be attributed to a shift towards infectious diseases with unusual clinical presentations that go along with the advances in medical treatment (e.g. organ transplantation). Moreover, as briefly mentioned below, all sophisticated diagnostic tools being used nowadays have limitations regarding sensitivity and/or specificity. For example, Kirch and Schafii [17] reported that CT scans and ultrasonography can yield false-positive and false-negative diagnoses for 6% to 9% of patients. Among other factors, these limitations may explain why pulmonary embolism as well as infarction and thrombosis (heart, mesenterium) are among the most often missed diagnoses in ICU patients [30], [31].

According to the current literature [3], [16], [17], the most often missed diagnoses are myocardial infarction and pulmonary embolism. In line with these data, our study shows cardiovascular diseases to account for the main part of class I discrepancies for all time points investigated.

We found significant declines of the class I discrepancy rates with respect to cardiovascular, neoplastic and pulmonary diseases as well as to internal medicine, and surgery. Indeed, all these observations most likely reflect the improvement of clinical ante-mortem diagnostics in the last decades. However, the class I discrepancy rate in patients coming from neurological wards nearly remained constant. Re-review of that special case group showed that, exactly as in the overall calculation, cardiovascular (e.g. myocardial infarction, pulmonary embolism) and neoplastic diseases (e.g. metastasized carcinoma, malignant lymphoma) were the most overseen diagnoses with relation to death.

In our opinion, an indifferent, or even dismissive attitude towards autopsy service in general, either by pathologists or clinicians is not conducive and ultimately counterproductive for the progress of clinical and scientific knowledge and the health care system in general [32], [33]. As already discussed previously by both clinicians and pathologists [2], [19], [34]–[37] there is a dual and uncontentious role for autopsies as a benchmark and to unravel diagnostic discrepancies, especially in times where economization and cost-effectiveness are on the top of the agenda [4], [38], [39]. The clinical non-forensic autopsy provides an economical and practical knowledge source that directly aids the physician [18, study initiated by the U.S. Department of Health and Human Services 2002], helps to describe novel diseases and is indispensable in physician teaching and additionally adds important epidemiological data to the mortality statistics [40] which often serve as a basis for decisions in health policy with far-reaching consequences. Furthermore, we are convinced, as other authors [3], [16], [18], [19], [24], that closer consultations between clinicians and pathologists before and after autopsy have already successfully reduced the diagnostic discrepancy rate over time and should still do in future.

Even if performed with the highest quality standard, ante-mortem diagnosis and clinical management have a multitude of inherent limitations (e.g. resolution power of ultrasound devices), which do influence clinical reasoning and decision making. This is why we, although widely used, avoided the term “diagnostic error” because an “error” interprets a diagnostic discrepancy negatively suggesting an individual clinical approach as solely causal for the discrepancy. The latter certainly affects discrepancies but is by no means the only and single cause.

We believe that the rise and continuous refinement of diagnostic tools and procedures as well as improving clinical management and knowledge serendipitously led and will lead to a steadily increasing number of patients whose diseases are recognized *intra vitam.* However, some patients remain with a higher individual probability of missing a certain diagnosis and we should direct our attention particularly to these [18], [41].

Thus, we tried to draw up a clinical profile for patients who have a high risk to receive a class I discrepancy. This may aid both pathologists and clinicians to identify patients, who may potentially benefit from exceptionally extensive and comprehensive diagnostics *intra vitam* and helps to focus on these autopsies that are most likely to uncover clinically relevant and death related but so far unrecognized diagnoses *post mortem.*

A methodic difficulty is connected with the simultaneous analysis of 67 subgroups for being at higher or lower risk for class I discrepancies. In this context, we refrained from correcting the p-values for multiple testing, because the sample size would be too small for a reasonable powered test. The situation is similar to subgroup analyses in clinical studies that are carried out without multiple testing corrections and should be considered as explorative [42].

Our risk-profile analysis visualized by heatmap-clustering singled out patient groups that are at potentially higher or lower risk for discrepant diagnoses. For instance, according to our data, patients with infectious diseases treated at a LAICU in a community hospital appear to have an increased risk for receiving a class I discrepancy. Re-review of that special case group revealed on the one hand overseen infections such as aspergillus and other fungal pneumonia, HIV-associated pneumocystis pneumonia, systemic nocardiosis or toxoplasmosis encephalitis, and on the other hand cases with, for example, undiagnosed pulmonary tuberculosis where sometimes disease-associated complications and/or other non-detected diseases finally led to death. It may be worthwhile to check whether similar data can be obtained from other studies and finally corroborate these data from retrospective studies in a prospective setting.

Interestingly, a comparison between the class I discrepancy rate of East Berlin (in the former GDR) and West Berlin (FRG) in 1988 revealed no statistically significant differences indicating a comparably high standard of clinical care at least in the Charité University Hospital (East Berlin) and the Virchow University Hospital of the Free University (West Berlin) on the verge of the Berlin wall break down. This is particularly remarkable since the autopsy rate at the Charité University Hospital (96.6%, East Berlin) was almost twice as high as in the Virchow University Hospital (44.5%, West Berlin) in 1988 (two years prior to German reunification).

## Supporting Information

Figure S1 **Class I discrepancy rates depending on the clinical subspeciality of the ward.**
(TIF)

Figure S2 **Development of class I discrepancies in six disease groups between 1988 and 2008.** The discrepancy rates were calculated by setting the number of cases with a class I discrepancy into relation to all patients with a diagnosis in a certain disease group. a) cardiovascular diseases (CV); b) neoplastic diseases (NP); c) pulmonary diseases (PU); d) gastrointestinal diseases (GI).
(TIF)

Figure S3 **Development of class I discrepancies between 1988 and 2008 stratified by the cause of death.** The discrepancy rates were calculated by setting the number of cases with a class I discrepancy into relation to all patients that died from a certain disease group. a) cardiovascular diseases (CV); b) neoplastic diseases (NP); c) pulmonary diseases (PU); d) gastrointestinal diseases (GI).
(TIF)
