# Supplementary figures and images for: Who Is at Risk for Diagnostic Discrepancies? Comparison of Pre- and Postmortal Diagnoses in 1800 Patients of 3 Medical Decades in East and West Berlin
Source: PLoS One. 2012 May 22;7(5):e37460. doi: 10.1371/journal.pone.0037460 (PMC3358345; doi:10.1371/journal.pone.0037460)

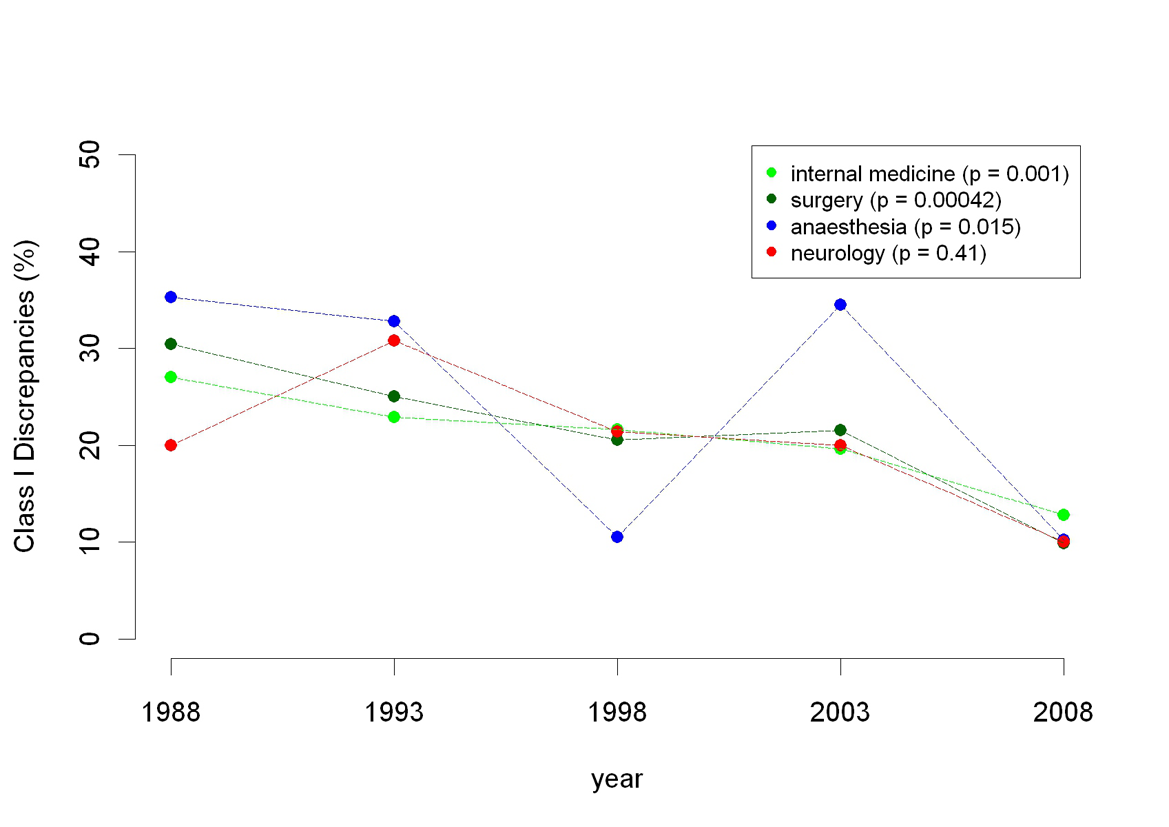

Supplement: Figure S1 — Class I discrepancy rates depending on the clinical subspeciality of the ward. (TIF) [file pone.0037460.s001.tif]

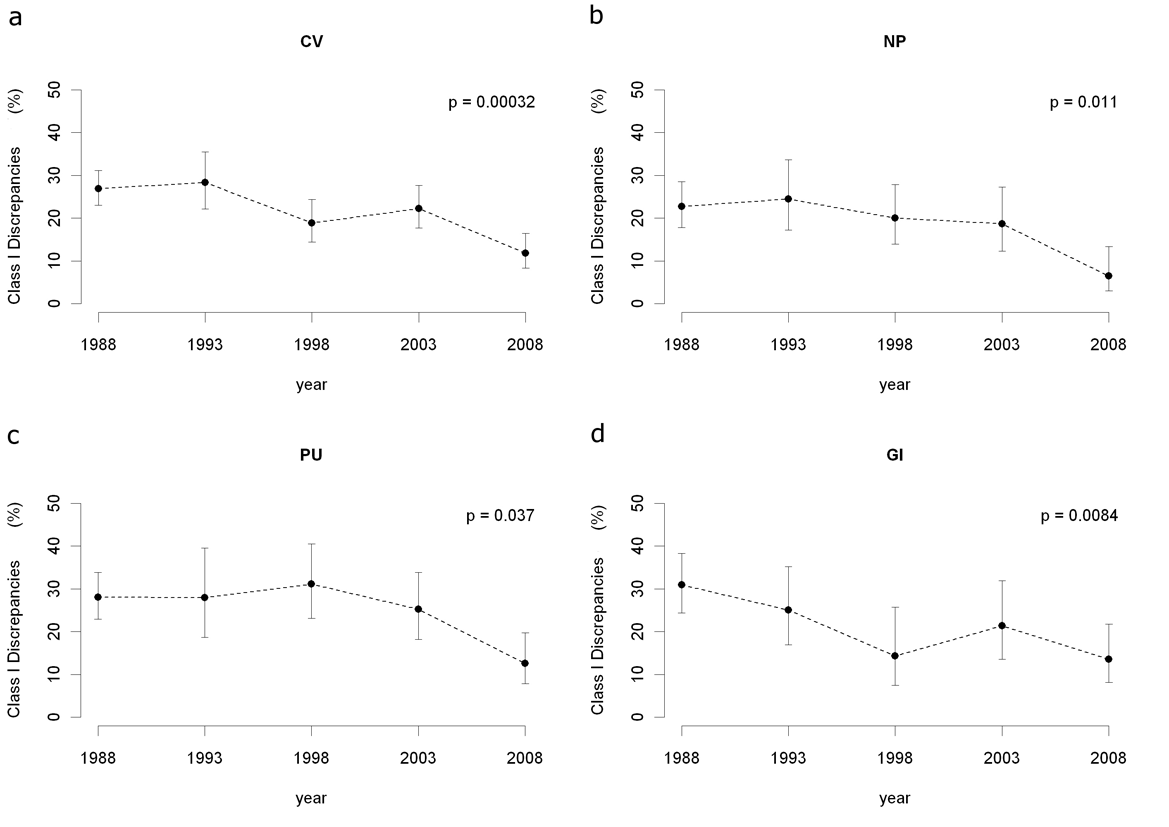

Supplement: Figure S2 — Development of class I discrepancies in six disease groups between 1988 and 2008. The discrepancy rates were calculated by setting the number of cases with a class I discrepancy into relation to all patients with a diagnosis in a certain disease group. a) cardiovascular diseases (CV); b) neoplastic diseases (NP); c) pulmonary diseases (PU); d) gastrointestinal diseases (GI). (TIF) [file pone.0037460.s002.tif]

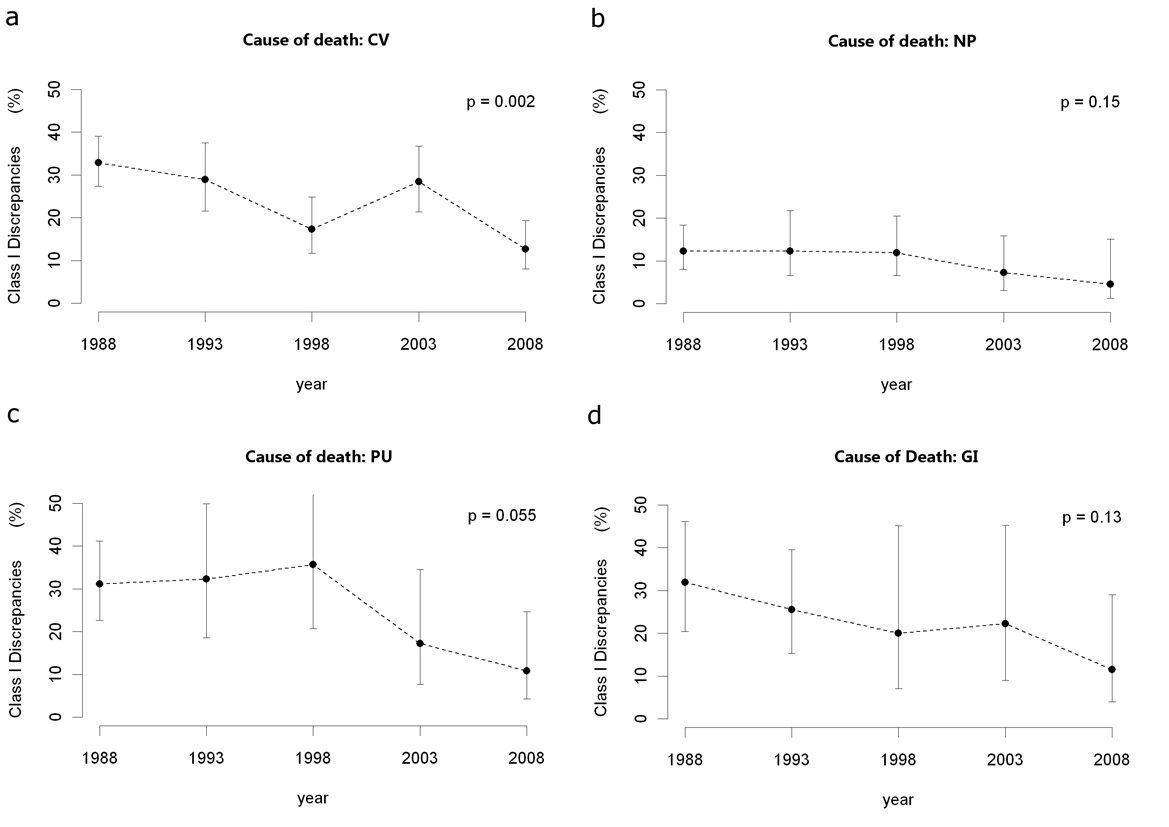

Supplement: Figure S3 — Development of class I discrepancies between 1988 and 2008 stratified by the cause of death. The discrepancy rates were calculated by setting the number of cases with a class I discrepancy into relation to all patients that died from a certain disease group. a) cardiovascular diseases (CV); b) neoplastic diseases (NP); c) pulmonary diseases (PU); d) gastrointestinal diseases (GI). (TIF) [file pone.0037460.s003.tif]
